# Risk analysis of the Unity 1.5 T MR‐Linac adapt‐to‐position workflow

**DOI:** 10.1002/acm2.13850

**Published:** 2022-11-21

**Authors:** Jiayi Liang, Paola Godoy Scripes, Neelam Tyagi, Ergys Subashi, Theresa Wunner, Nicolas Cote, Ching‐Yun Chan, Angela Ng, Victoria Brennan, Kaveh Zakeri, Cassandra Wildberger, James Mechalakos

**Affiliations:** ^1^ Department of Medical Physics Memorial Sloan Kettering Cancer Center New York NY USA; ^2^ Department of Radiation Oncology Memorial Sloan Kettering Cancer Center New York NY USA

**Keywords:** adapt to position, ATP, failure modes and effects analysis, FMEA, MR‐guided radiation therapy, MR‐Linac

## Abstract

**Background and Purpose:**

Newer technologies allow for daily treatment adaptation, providing the ability to account for setup variations and organ motion but comes at the cost of increasing the treatment workflow complexity. One such technology is the adapt‐to‐position (ATP) workflow on the Unity MR‐Linac. Prospective risk assessment of a new workflow allows clinics to catch errors before they occur, especially for processes that include novel and unfamiliar steps.

**Methods:**

As part of a quality management program, failure modes and effects analysis was performed on the ATP treatment workflow following the recommendations of AAPM’s Task Group 100. A multidisciplinary team was formed to identify and evaluate failure modes for all the steps taken during a daily treatment workflow. Failure modes of high severity and overall score were isolated and addressed.

**Results:**

Mitigations were determined for high‐ranking failure modes and implemented into the clinic. High‐ranking failure modes existed in all steps of the workflow. Failure modes were then rescored to evaluate the effectiveness of the mitigations.

**Conclusion:**

Failure modes and effects analysis on the Unity MR‐Linac highlighted areas in the ATP workflow that could be prone to failures and allowed our clinic to change the process to be more robust.

## 1 INTRODUCTION

The Elekta Unity 1.5 T MR‐Linac (MRL) couples a 7 MV flattening‐filter‐free photon beam with a 1.5 Tesla (T) MRI scanner (Philips Healthcare, Best, the Netherlands). This machine can adapt radiotherapy plans daily using high‐resolution MRI images. Treatment planning is done in Monaco (Elekta AB, Stockholm, Sweden) and the record and verify system is MOSAIQ (Elekta AB, Stockholm, Sweden). Since the patient receives a recalculated plan every fraction, scrutiny of the workflow is important to ensure safe and accurate dose delivery.

The Unity MRL offers two modes of plan adaptation. The adapt‐to‐position (ATP) workflow is a virtual couch shift that shifts the fields to match to the position of the target for the day, analogous to a table shift on a conventional linear accelerator, with the option to optimize existing segment shapes and their weights with the goal of reproducing target dose. The more involved adapt‐to‐shape (ATS) workflow performs a full replan of the patient's treatment, allowing the user to recontour structures to match the anatomy of the day and adjust target goals and constraints. While there are benefits to both methods, the steps taken change depending on which mode of adaptation is used.

Regardless of the method, plan adaptation on the MRL is a team effort. Therapists set up and treat the patient. MR technicians acquire appropriate sequences at multiple time points during the treatment appointment. Dosimetrists or trained therapists (planners) perform fusions and create plan adaptations, making judgement calls on whether the selected adaptation method is appropriate given the patient's positioning for the day. Physicists perform dosimetric checks on the plan prior to the first fraction and on the adapted plan before treatment. Physicians will recontour the target and key organs at risk (OAR) for ATS treatments and assist in the evaluation of image fusions.

Since the workflow requires multiple different tasks from different members of the clinical team, it is important to evaluate the workflow for potential failure modes. Risk analysis, following the guidelines of Task Group (TG)‐100^1^ was performed on the ATP workflow for the Unity MRL (a subsequent study will address ATS). A process map was first created through multiple observations and discussions amongst team members. Then a failure modes and effects analysis (FMEA) was performed using that process map to identify sources of error. The identified failure modes subsequently helped to facilitate changes to the present workflow to circumvent the points where mistakes could occur.

## 2 METHODS/MATERIALS

A team was created to follow the TG‐100 methodology for risk analysis. This team consisted of 14 members and included 2 therapists, 1 MR technician, 2 physicians, 3 dosimetrists, and 6 physicists who actively treat patients on the Unity. This risk analysis was done while the clinic used Monaco version 5.40 and MOSAIQ version 2.65 but has considered the completion plan workflow changes that were implemented in Monaco version 5.51.11 and MOSAIQ version 2.83.

A process map was first created to codify the various steps of the ATP workflow at our institution. This process map concentrated only on the steps that were taken on the treatment day. The workflow for pretreatment activities, such as simulation, contouring, or initial reference plan generation, were not evaluated since those workflows continued to follow standard guidelines of the department and would be better addressed with their own analysis. The process map started with preparations needed to treat the patient for the day, continued through setup, image acquisition, adaptive plan generation, and ended with post treatment activities. The initial draft of the process map was created by the two team leaders. Then the rest of the team reviewed the process map and made changes or additions as needed. Team members were encouraged to review the entire process map but especially the process step of their expertise.

Once the process map was finalized, failure modes were identified for each step using a prospective approach which relied on the team's knowledge of potential failures^1^ as well as a retrospective approach which drew on near misses or events which had already occurred. To facilitate this, the entire team met for a series of “TG‐100 Retreats” which lasted half a day. In addition to the failure modes, potential causes of failures were also brainstormed. For all identified failure modes and causes of failures, it was assumed that no quality assurance (QA) procedures were in place per TG‐100 guidelines.^1^

After the failure modes were identified, the results were compiled into the FMEA scoring template provided by the American Association of Physicists in Medicine (AAPM) Repository of TG‐100 Tools.^2^ This document was then handed out to the entire team for individual scoring. Each potential cause of failure was scored for its likelihood of occurrence (O), the potential severity (S) if it did happen, and the detectability (D) of the risk. The scores for each individual category were then multiplied to obtain the risk priority number (RPN).^1^ A slight alteration of the TG‐100 methodology was made for simplicity in that each category was scored from 1 to 5 rather than 1 to 10. For likelihood of occurrence, 1 indicated that the failure was unlikely while 5 indicated that it is highly likely. For severity, 1 indicated that it would be an inconvenience if the failure was to occur while 5 indicated the result would be catastrophic or lethal. For detectability, 1 represented that the failure would be obvious and easily detected, while 5 would mean there is a great likelihood to go undetected.^2^ To support the team in the scoring process which lasted several weeks, the team leaders held TG‐100 “office hours” where they were available virtually for 1 h each week to answer questions or provide clarification.^3^

Once all the scores were returned to the team leaders, failures were sorted following recommendations of TG‐100. Failures with the top 20% highest RPN scores based on both average and median scores and top 20% highest severity scores were isolated for analysis. Another all‐staff retreat was scheduled for the team to review the highest scoring failure modes and to brainstorm mitigations to circumvent the failures. Staff whose role aligned with the subprocess which contained the failure mode were especially suited for selecting the intervention. For example, therapists were most suited to discuss patient identification and setup. Recommendations from the team were then presented during a department‐wide MRL meeting so that any implementation concerns could be addressed. Team members were then asked to rescore the high‐ranking failure modes with consideration of the mitigations to evaluate their effectiveness.

In our clinic, there are three use cases for ATP: (1) a treatment that is intended to be ATP only, which at the writing of this document, is implemented regularly only for the treatment for head and neck cancers; (2) ATP that is done as part of the ATS cases to account for small shifts that occurred during the planning process without the need to recontour; (3) completion plans which are generated to continue a partial treatment with imaging. The final process map consisted of eight main stages which are described below:

1. Preparation: This consists of all steps taken on the day of treatment to prepare the room and patient. Vendor software is launched prior to bringing the patient into the treatment room. This includes MOSAIQ record and verify and treatment session manager, Monaco treatment planning system, the screen‐sharing software WebEx (Cisco, San Jose, CA) (team members used WebEx so as not to crowd around the same PC), and the log‐file analysis software LinacView (Standard Imaging, Inc., Middleton, WI). Approval of necessary documentation such as the previous day's ATP plan if applicable is also checked by the therapist. When the patient arrives, front desk staff will ask for the patient's name and birthdate, provide an identification wristband and an MRI questionnaire which is filled out by the patient while waiting. When called, the patient will then have their name and birthday rechecked both verbally with the therapist and visually with the wristband. The patient is then escorted into MRI zone II to fully undress and change into a gown. While the patient changes, the therapists review the patient's MRI questionnaire and complete the daily assessment in MOSAIQ following the questionnaire. If there are no concerns after reviewing the MRI questionnaire, the patient is brought into zone III and scanned for metal using a metal detection wand before being escorted to zone IV.
  - Although rare in ATP cases, should the patient require contrast, the procedure varies depending on the type of contrast. For contrasts that can be administered ahead of time, it is done while the patient is in the waiting room (zone I). For contrasts that need to be delivered just before imaging, it is done in the treatment room (zone IV) with appropriate MRI‐safe equipment.
2. Positioning and imaging: The therapists set up the patient to the appropriate table index and with appropriate immobilization devices as per the simulation document. This takes approximately 5 min, not including the time it takes to give contrast in the treatment room. Then, the MR technician acquires an initial 3D MRI image for positioning/adaptation. The sequence used in our clinic takes 3 min and 30 s to acquire. When the 3D image finishes reconstruction, the image is manually sent by the MR technician from the scanner to online Monaco. The transfer time is less than a minute.
3. Fusion/preoptimization: The planner, who sits by the control console in zone III, selects the reference plan/image to be used for adaptation to today's treatment; if multiple options are available, it is picked based on treatment notes from prior fractions. The image of the day is imported and rigidly fused to the reference plan image. This takes approximately 4 min.
  - For ATP cases done during ATS, such as hypofractionated prostate treatments, the ATP workflow would begin here. Presumably, a verification image was just taken, and evaluation of this image resulted in the decision to further adapt the plan using ATP. It is important that the selected reference plan is the ATS plan that was just generated so that correct virtual couch shift is applied.
  - For cases where delivery is resumed after a partial treatment, the only plan available for selection is the completion plan.
4. Optimization: The mode of adaptation and the method of optimization are selected by the planner, and the optimization is started. The goal of ATP reoptimization after a virtual couch shift is to reproduce target goal dose. During the calculation time, electron density on the MR is checked by the planner and physicist and the real‐time display of isodose lines is monitored. Just before the optimization finishes, the MR technician takes a verification MR image. The optimization process takes approximately 3 min.
  - For completion plans, the only allowed mode of adaption is ATP and the optimization method cannot be changed from the default setting of “Reproduce Goal Dose.” The standard ATP option of “Improve Target Dose” would cause the optimizer to attempt to achieve the full prescription dose instead of the intended partial dose and is thus disabled by Monaco.
5. Plan review and approval: The verification image is imported and evaluated by the planner and physicist for any potential motion. Then, the final isodose lines and DVH statistics are reviewed. Plan adjustments may be made at this time depending on the amount of motion that occurred. If no adjustments are needed the plan is approved and automatically sent to MOSAIQ for treatment.
6. Plan check: The plan is checked by the physicist prior to delivery to ensure data transfer integrity. Plan parameters between Monaco and MOSAIQ are compared using in‐house software. This process takes less than 2 min. Motion monitoring (MM) using three orthogonal cine MRs is started, by the therapist, during this time as a real‐time cine‐MR display of patient motion.
7. Treatment: The therapist selects the newly adapted plan for delivery. Independent monitor unit (MU) calculation may be performed by the physicist after beaming on for conventionally fractionated ATP cases to reduce waiting time. MM is on the entire time except in rare cases where a post treatment MRI is indicated. For these cases, MM is stopped near the end of delivery to allow time to acquire the post treatment MRI as treatment completes.
8. Post treatment: Treatment is recorded by the therapist and the planner compiles a plan report with fusion and dose statistics for the physician to approve in ARIA Dynamic Documents (MOSAIQ is only used as a record and verify system for the MR‐Linac at our institution, all documentation is stored in ARIA as this is the primary radiation oncology electronic medical record system for our facility). The treatment log‐files are checked against the plan using the LinacView application and the results are reviewed by the physicist. If taken, the planner will evaluate the post treatment MRI and notify the physician of any significant changes; dose may be recalculated on the post treatment MRI to estimate organ doses for those proximal to high dose regions. All images are saved to a backup server.

We had initially mentioned that there were three alternative ATP workflows being considered. The workflow shown above corresponds to the use case in which ATP is the intended treatment. The other two use cases, namely that in which an ATS plan is done but an ATP is done to adjust due to patient motion during the online planning process, as well as the completion plan workflow, can also be represented by the above workflow. In the case of the ATS workflow, the ATP plan is created between steps 2 and 3. The ATS workflow will be covered in a later report. In the case of the completion plan workflow, the completion plan is created offline and is chosen as the reference plan, therefore, these other two use cases only differ in the origin of the reference plan chosen for the ATP process. The potential failure modes for creation of the completion plan are mentioned briefly as a separate subsection of the results.

## 3 RESULTS

The workflow required on the Unity MR‐Linac is very different from that of a “standard” non‐MR‐guided linear accelerator in our clinic. In response, an online reference form was created to help guide medical physics team members through the necessary steps. This form contains all the steps necessary for treatment in sequential order and was intended to be used by the planner during the treatment workflow. It also includes patient‐specific information, such as treatment prescription and target margins. Since the treating team varies day by day, the form also serves as a document for teammates to leave additional comments about the treatment, including the reference plan name that should be used for the next fraction. Although it was created before this risk analysis was performed, following along this reference form could potentially help to mitigate several failure modes across the entire process map, including those that were below the 20th percentile. But because of the sheer number of steps, usage of it is better suited for new staff or to review before the patient is on the treatment table.

**FIGURE 1:**
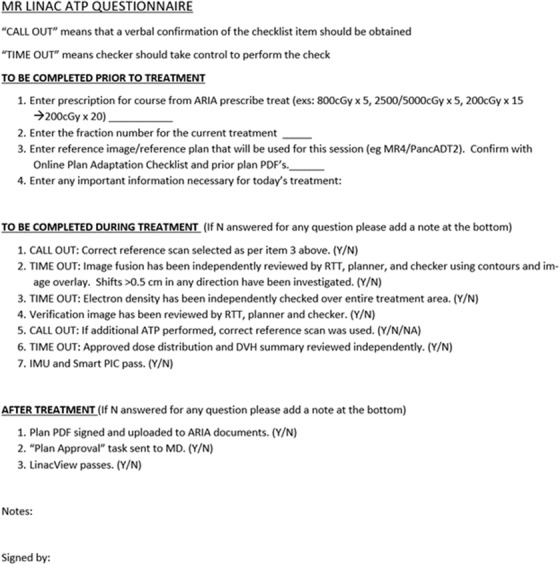
ATP checklist used before, during, and after a patient's treatment fraction.

Since this risk analysis was performed, a shorter online checklist was created. This checklist includes only the most important reminders in a concise format and better adheres to the recommendation of Medical Physics Practice Guideline 4.a for the development of checklists.^4^ It is embedded with “yes”/“no” or fill‐in‐the‐blank answer prompts (see Figure 1). The intended user, the physicist, will fill out this document before, during, and after the treatment fraction for each fraction of each patient's treatment. The checklist is used to help mitigate several high‐ranking failure modes.

**FIGURE 2:**
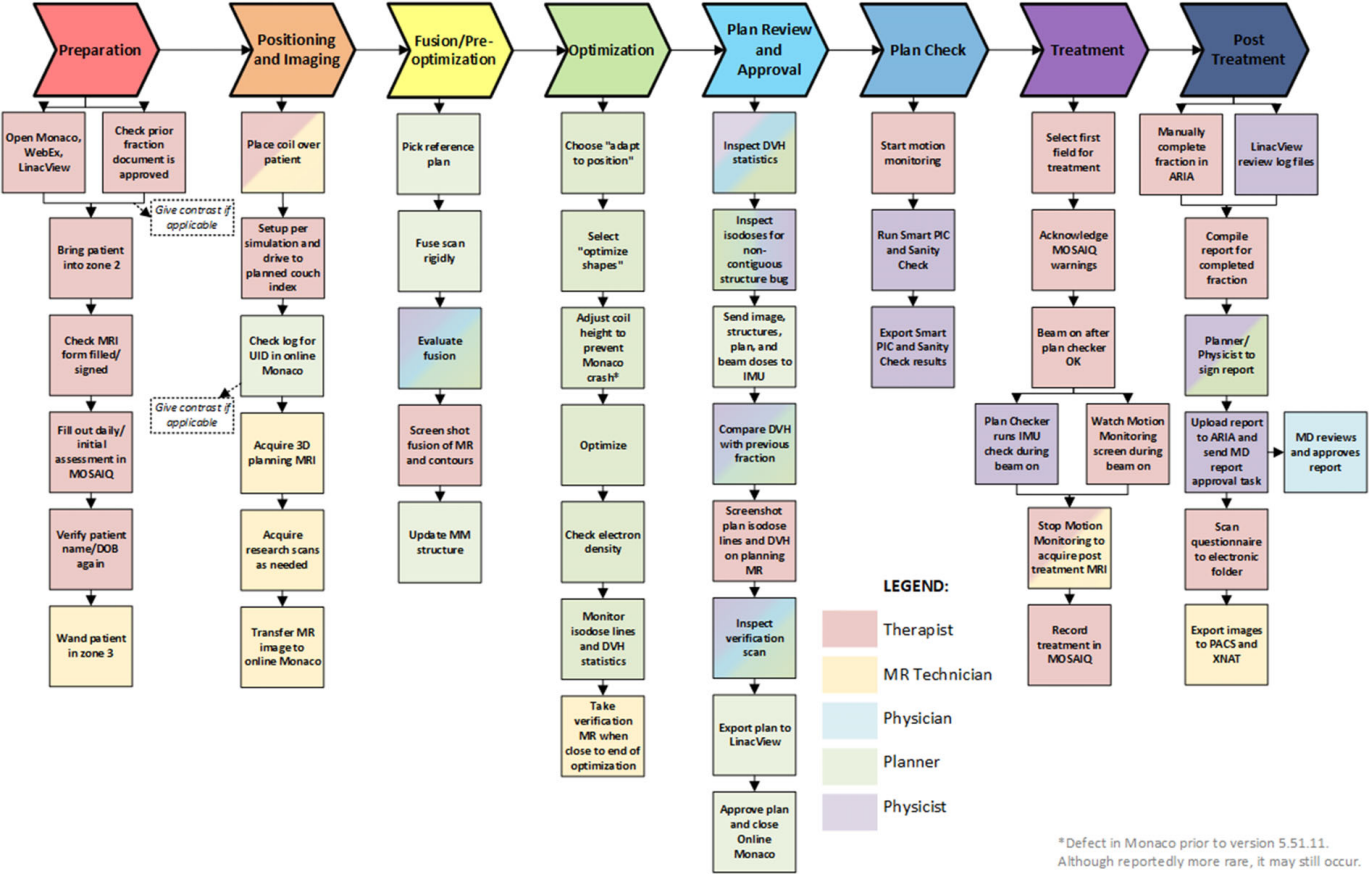
Process map of the adapt‐to‐position workflow. The colors of each box indicate the responsible staff member for that step. Multiple colors indicate that multiple staff may be responsible in completing that step.

The main ATP process map is shown in Figure 2. Two hundred and sixteen failure modes were identified and scored assuming no mitigations were in place. Since the physician's role in ATP was limited to the ATS cases that needed ATP due to minor shifts, the two physicians only scored the failure modes of steps they were familiar with. The other members of the team scored all failure modes since they actively engaged in all parts of the workflow. After sorting the failure modes based on average RPN score, median RPN score, and severity, a total of 45 failure modes were highlighted for mitigation strategies.

Mitigation strategies were discussed amongst the team members. When possible, automated checks were included, but when it was not possible, having redundancy within the workflow helped to maximize the likelihood of catching the failure. For each step in the ATP process map, selected failure modes and mitigations are discussed below. A full list of the high‐ranking failure modes and mitigations are shown in Table 1.

**TABLE 1:** List of failure modes and mitigations for the ATP workflow on the Unity

Step	Process map step name	Potential failure mode(s)	Potential causes of failure	Mitigation(s) for failure mode
**1**	**Preparation**			
	Bring patient into room	Wrong patient	Name and birthday not verified	(1) Front desk checks patient name/birthday and gives wrist band; (2) therapists reverify name/birthday, and (3) confirm with wristbands given at front desk
		Wrong plan loaded on MOSAIQ	Photo check missed	Therapists have tablet that view the MOSAIQ computer which has patient photo
	MRI questionnaire form filled and signed (patient, therapist, witness)	Questionnaire filled out incorrectly; patient contains ferromagnetic material	Patient did not read thoroughly	(1) Tablet to fill out MOSAIQ assessment with patient; (2) daily implant email for MRI patients
			Patient forgot about implant	(1) Daily automated implant email for MRI patients; (2) therapist to question scars
	Daily/initial assessment in MOSAIQ	Filled out form incorrectly	Questionnaire incorrect so wrong information put in assessment as a result	(1) Tablet to fill out MOSAIQ assessment with patient; 2) daily implant email for MRI patients
			Therapist mistake when filling out assessment	Saying “yes” to implant or pregnant shows up red in MOSAIQ and MRI eligibility is not met so treatment will be prevented
	Verify patient name and birthday with patient again	Second therapist did not verify twice	Thought first therapist verified	It is a good check to have but patient will have had three other verifications of id (front desk, first therapist, photo check)
	Wand patient in zone III	Wand malfunctions	Electronics error	(1) Have backup wand at MRI sim and (2) in‐room metal detectors (on wall)
		Wanding not complete	Did not wand all over	Must complete wand training module before wanding patient
			Move wand too fast	Must complete wand training module before wanding patient
		Wand not on	Thought on but it is not	Use wand only with sound on
		Did not wand	Unaware of policy	(1) MRI training provides understanding dangers of ferromagnetic materials; (2) patients are in gowns so chance of carrying metal on body is low
			Forgot	(1) In‐room metal detectors should go off; (2) patients are in gowns so chance of carrying metal on body is low
**2**	**Positioning and imaging**			
	Setup per simulation and drive to couch index per plan	Patient has moved in mold such that fields are deliverable (small field)	Patient discomfort	Verification scan will be thoroughly reviewed by planner and physicists to catch motion
		Couch goes to wrong location	Board level not entered correctly in sim	(1) Monaco does not allow automated shifts >5 cm; (2) department policy requires all shifts >0.5 cm to be rechecked
	Take other sequences as needed for research	Motion monitoring stopped early to accommodate research scan	Unaware of policy	Training for designated MM watcher should include knowledge that stopping mm is only allowed for post MRI image
	Transfer from MARLIN to online using “Monaco” destination	Wrong scan sent	Did not check time or date	MRI tech to “call‐out” patient name, image date + time and MRI sequence; planner will verbally confirm
**3**	**Fusion/preoptimization**			
	Decide which reference plan to used based on prior fraction notes as applicable	Wrong plan is chosen	Entered correctly in checklist but wrong plan chosen in the dropdown (ATP only treatment)	(1) Planner to “call‐out” MRI number and plan name/number and therapist and physicist to verify call‐out and on‐screen; (2) smart PIC to check if plan is based on most recent ATS
			Entered correctly in checklist but wrong plan chosen in the dropdown (ATP after ATS for prostate)	(1) therapists should update excel sheet with ATS plan number before proceeding to ATP; (2) planner to “call‐out” MRI number and plan name/number and therapist and physicist to verify call‐out and on‐screen; (3) Smart PIC to check if plan is based on most recent ATS
			Reference plan name that should be used was entered incorrectly/vague on online checklist	Treating physicist should independently determine which reference plan to use beforehand
	Select appropriate reference plan name in online Monaco under “reference plan”	After prostate ATS and ATP is now needed, the wrong scan is selected	Mistakenly selected wrong plan	(1) Planner to “call‐out” MRI number and plan name/number and therapist and physicist to verify call‐out and on‐screen; (2) smart PIC to check if plan is based on most recent ATS
	Fuse scan rigidly	Fused incorrectly	Fusion controls used improperly	Planner and physicist to *independently* review fusion
	Evaluate fusion	Primary and secondary scans mixed up during registration	Not clarified by planner	Always double check “show images” window to see which image is in active view
		Patient weight loss causes significant difference in fusion	Patient weight change	Call MD if overall change is 1 cm or more, as it may require ATS instead
		Better to match on non‐bone structure but matched on bone	Matched on wrong anatomic area thinking it was correct	Out “fuse on” information in online checklist
**4**	**Optimization**			
	Optimize	Stray target contours leading to stray dose	Did not thoroughly evaluate contours in reference plan	Cannot make changes in ATP so need to address this during initial offline plan check
	Check ED under grid type > electron density	ED is not correct on reference scan	Planner did not check thoroughly, or wrong layer order or ED not assigned	(1) ED is double checked online: all planners (including therapist planners) are trained to expect max of 1.8 unless there is an implant; (2) review of ED assignment should include entire image in all three planes
	Take verification MRI	Did not take and patient moved	Forgot or unaware of policy	Online checklist states order of steps and includes taking the verification MRI
**5**	**Plan review and approval**			
	Inspect DVH statistics (cursory)	Constraints incorrect for PTV	Incorrect because multiphase plan not scaled correctly	Cannot change constraints for ATP: checked thoroughly during initial offline plan check
			Constraints incorrect because template changed in prior fraction	(1) Online checklist states which reference plan to use; (2) physicist also independently determines this before treatment; (3) planner will use call‐out procedure to ensure selection is correct
		Constraints incorrect for critical organ (limit)	Incorrect because multiphase plan not scaled correctly	Cannot change constraints for ATP: checked thoroughly during initial offline plan check
		Inspect isodoses for bug in non‐contiguous structures	Did not check	(1) Planner and physicist should review dose together; (2) cannot change contours in ATP, so must be checked during initial offline plan check
			Unaware of policy	(1) Instructions are in online checklist to review plan before approval; (2) cannot change contours in ATP, so must be checked during initial offline plan check
	Inspect verification scan by toggling between MRI old and new MRI (in drop down bar)	Should have done ATS because of patient motion but did ATP	Did not review thoroughly verification images	Planner and physicist should *independently* review verification images
			Accidentally reviewed the wrong images (not new MRI)	Always double check “show images” window to see which image is in active view
		Gas bubble pushing into PTV prompts second ATS which is missed	Did not review thoroughly verification images	Planner and physicist should *independently* review verification images
	Approve plan and close Monaco	Transfer incorrect from Monaco to MOSAIQ	Control points or mu does not match; partial plan transfer; gantry does not match	Smart PIC will catch any of these by comparing Monaco to MOSAIQ
**6**	**Plan check**			
	Run smart PIC and sanity check	Did not run PIC or sanity check	Forgot	Add check to IMU script to make sure PIC passes (acts as reminder to run PIC too)
**7**	**Treatment**			
	Run IMU	IMU returns a false positive	Incorrect ED	ED is checked during plan optimization; planners/therapists are trained to know the expected range and to review ED in all three planes along the entire image
			Dose calculation issue	Do arc check after treatment
	Watch MM	Did not watch MM	Other issues to attend to	(1) Will have designated MM watcher who sole duty is to watch the MM screen; (2) implement action levels of what to do if there is motion
			Unaware of policy	(1) Update online checklist to include a “watch MM” step; (2) assign roles prior to patient treatment of who does what
		Contour on MM not representative of oar motion (i.e., wrong slice)	Center of contour not ideal	Cannot change contours in ATP: check center of MM structure during initial offline plan check or most recent ATS fraction
**8**	**Post treatment**			
	MD signs report in aria	Miss bad fusion	Did not review thoroughly	Planner and physicist will independently review fusion so this should not happen
		Miss bad DVH	Did not review thoroughly	Planner and physicist will review dose prior to treating so this should not happen

Abbreviations: ATP, adapt‐to‐position; ATS, adapt‐to‐shape; DVH, dose volume histogram; ED, electron density; IMU, independent monitor unit verification; MM, motion monitoring, MRI, magnetic resonance imaging; PIC, plan integrity check; PTV, planning target volume.

### 3.1 Preparation

One of the highest scoring failure modes was treatment with the wrong plan. This failure mode scored in the top 10th percentile for highest median RPN score and was in the top 10 for highest severity. This failure mode could be due to either bringing the wrong patient to the treatment room or having the wrong patient loaded in MOSAIQ.

While scoring of failure modes did not account for current QA policies, addressing this failure mode relies on procedures already in place. When patients check in with the front desk, staff will confirm the patient's name and date of birth. Then, they are instructed to wear a wrist band with their name, date of birth, and medical record number. The therapists will check the information on the wrist band in addition to reverifying name and date of birth with the patient.

Similarly, treating a patient with ferromagnetic material in his or her body also scored in the top 10th percentile for highest median RPN score. When patients check in with the front desk, they are handed an MRI questionnaire to detail any surgeries and implants they previously received. When the patient is called for treatment, the therapist will take this questionnaire to fill out the daily MOSAIQ assessment form which is required for treatment. The failure mode that arises from this step is that the patient may forget to acknowledge an implant that could be dangerous when exposed to a high magnetic field. Since therapists fill out the MOSAIQ form without the patient present, the detectability is reduced.

A few procedures already in place also act as mitigations to this failure mode. In addition to using the patient questionnaire to obtain information about ferromagnetic material, an automated email is sent daily, listing all patients scheduled to receive an MRI with the radiation oncology department. Implant data for this email is pulled from the institution‐wide electronic medical record system; at any point during a patient's care, regardless of the department, if implant information is entered into the system, that information is subsequently available to all caretakers. As one of the mitigations used to prevent the treatment of patients with ferromagnetic implants, MRL patients were included in the daily automated email. In addition to this email, therapists also question patients about any visible scars.

From the mitigation meeting with the MRL team, a tablet was recommended so that the therapist could fill out the MOSAIQ questionnaire with the patient present. With the tablet, the therapists can review the important parts of the questionnaire with the patient; it also means that a picture of the patient, taken during initial patient registration, would also be at hand for identification. Having the tablet view and control the treatment computer connected to the MRL would also act as a secondary check to ensure the correct patient is loaded for treatment, reducing the chances of treating the wrong patient's plan. If an additional purchase is not feasible, the recommendation is still to complete the questionnaire with the patient present such as a workstation nearby the changing area, or at the very least, with the patient in zone III. Therapists also reconfirm that the patient does not have any implants and is not carrying metal when they wand the patient.

Another failure mode that ranked within the 20th percentile of overall highest RPN score relates to the wanding procedure done before the patient enters zone IV. One failure mode is that the wand malfunctions and does not alert the staff of ferromagnetic material on the patient. The other failure mode is that staff does not wand the patient thoroughly enough, either not scanning all parts of the patient or scanning too fast for the detector to work.

Although these failure modes came up during this risk analysis, the basis for these failure modes is not specific to the MRL and relates more to general MR safety. Ferromagnetic materials that are not secured can become a projectile danger to the patient and staff, especially larger patient transport objects such as wheelchairs and stretchers.^5^ ACR guidance documents recommend always restricting entry into zone IV by means of keeping the door closed or other easily adjusted barriers when the MR door must remain open.^6^ Implanted non‐compatible medical devices may pose significant dangers to the patient in the presence of a high magnetic field. Forces from the magnetic field may displace implanted devices. Cardiac devices may malfunction and cause cardiac events or even patient death. Non‐compatible metals may burn the patient internally.^7^ ACR recommends a “full‐stop and final check” to ensure that all necessary safety screenings of the patient, equipment, and personnel are performed prior to entering zone IV. External burns are also of concern, especially from clothing that contain impurities. ACR recommendation is to have the patient wear MR‐safe gowns.^6^

The wanding procedure helps to prevent the entry of non‐MR‐safe materials into zone IV. The mitigation for wand failures already exists in our current workflow. To ensure proper wand function, staff complete wand training prior to use and check that the wand lights up and test the wand on themselves. A backup wand is also available in case of malfunction. To speak to general MR safety, our clinic follows ACR MR safety guidelines. Patients are told to fully undress and change into a gown, minimizing the chance of metal being kept on their person. Redundant metal detectors are also placed on the walls in zone III, which further minimizes the chances of ferromagnetic material being brought into the treatment room. As an additional mitigation, the risk analysis group also suggests using the metal detection wand with the sound on when performing the wand check for metals and turning the sound on for the redundant wall detectors, reducing the chance of missing notification of metal detection. Our clinic also requires staff members to complete two MR safety training modules every year to maintain competency.

### 3.2 Positioning and imaging

To position the patient, the therapists will set up the patient to align their isocenter tattoos, received during the simulation appointment, to the same board level used at simulation aligned with the appropriate MR‐Linac couch index. The board level and couch index values are manually transcribed by the simulation therapist into a form. The planner also manually enters the couch index value in Monaco to set as the isocenter for the plan. Transcription errors from either step were a high‐ranking failure mode.

The mitigation for this failure mode is partially integrated in online Monaco, as the system will not allow for automated shifts greater than 5 cm. For our department, we also instituted a policy that for shifts greater than 0.5 cm, the therapists must investigate what is causing the discrepancy. This may include going back into the room to recheck the patient's positioning.

In the imaging step, a high‐ranking failure mode was sending the wrong scan to the treatment planning system. The standard vendor workflow has the image being automatically sent to online Monaco. It was a decision made in our clinic to deviate from the vendor standard option so that we could have more flexibility in our workflows and allow us to acquire multiple MR sequences. In MARLIN (Philips Healthcare, Best, the Netherlands), the MR scanner attached to the MRL, the software displays different series as an expandable folder that can be sorted by patient name and scan date and time. A patient who has received more than one series of MR scans will have a folder that corresponds to each series; if multiple series were taken that day, the patient would have multiple folders all with the same date. The MR sequences taken for each fraction is usually the same, so the only differentiating factor between the different folders would be the scan date and time. Thus, a reasonable concern most team members had was that the wrong scan would be sent for adaptation. It could be especially concerning if the patient had to be taken off the table after imaging and then set up again later, as this would create a second folder with the same scan date.

To try to circumvent this, the suggested mitigation was to implement a “call‐out” policy. The MR technician would verbally call out the patient's name, scan name, date, and time before they send the image to Monaco. The planner should then confirm that the appropriate scan is being selected and verbally acknowledge the call.

### 3.3 Fusion and preoptimization

After importing the image of the day to Monaco, the next step is to select the reference MR and plan. This is an important part of the workflow because in ATP, the new plan will be adapted to account for virtual couch shift and will try to reproduce the target goal dose distribution in the reference plan. Choosing the wrong reference plan could mean that any volume or dose changes that occurred in a prior ATS fraction would not be carried over to the current ATP plan. This failure mode scored in the 10th percentile for highest median RPN score. Depending on how many fractions the patient was previously treated, the list of approved reference plans could be very long. All plans created by adaptation have the same name except for a difference in the trailing number. Also, all plans previously approved, including adapted ones, are available to use as a reference plan, making it easy to select the wrong plan.

The current QA process to ensure the correct reference plan is selected and uses the online checklist as a venue for commentary between different planners. After contour adjustments made during an ATS treatment, the planner would note the adapted plan number which would be the reference plan for all subsequent ATP fractions until the next ATS. This annotation of the checklist typically occurs after treatment since the online Monaco workstation only has local network access.

However, to not rely on only one means of QA for such an important step of the workflow, a few mitigations were proposed for this failure mode. First, the ARIA questionnaire shown in Figure 1 was implemented such that the secondary physicist would be required to independently determine which reference plan should be selected prior to treatment. Most often, it would be the most recent ATS fraction where contours were updated by the physician. This also helps to reduce the cases where the wrong reference plan is used due to transcription errors. Second, when choosing the reference plan on the day of treatment, the planner would also verbally call out the plan name and the associated MR number, like the “call‐out” procedure performed by the MR technician in an earlier step. The role of the secondary physicist would then be to verify the plan name and MR number against their form. Finally, an automated second check was recommended that verifies if the adapted plan of the day is based on the most recent ATS plan. It would function as follows: after the adapted plan is approved and sent to MOSAIQ, existing in‐house software compares the adapted plan to the reference plan. Since all ATP plans are attached to the reference image, the software would verify that plan is attached to the most recent ATS fraction. While it is possible the treatment of the day could intentionally not be based on the most recent ATS, this automated check offers a quick sanity check.

Once the appropriate reference MR and plan is selected, a fusion between the reference image and the MR of the day is needed. The high‐ranking failure mode the team identified in this stage was either accepting a poor‐quality fusion or not properly evaluating the fusion. By not thoroughly reviewing the images, the team's concern was that patient motion or air regions near high dose regions would not be properly considered. It is also possible to review the wrong image. During fusion review, the keyboard shortcuts “home” and “end” can be used to toggle between the current and reference images. Other than a small text area at the top of the window, there is no other on‐screen display of which image is currently being viewed. Since the MR sequence used for planning is the same every day, it is very difficult to differentiate between two dates. The keyboard shortcuts serve as an additional source of error, as it could be possible to toggle one of those keys instead of the “page up” or “page down” key used to scroll through the image. This failure mode occurred as a near miss in our clinic prior to the risk assessment analysis. Since there are no other indications of the image name or date being viewed, the planner thought the fusion was very good and did not realize until the plan review phase that the wrong image was used for fusion analysis.

The suggested mitigation for this failure mode was to have a more involved fusion review process. Both the planner and the physicist should independently review the fusion, making sure that they evaluate the images slowly and thoroughly. Additionally, both parties should always double check the “show images” window to ensure that the correct MR image is in view. The independent fusion evaluation was incorporated into the physicist questionnaire described in the previous paragraph (Figure 1).

### 3.4 Optimization

After the fusion, a new plan is created to account for virtual couch shift. Before selecting the optimization option, the user may want to change the adapted plan's coil height. There is a known defect in Monaco where the system may crash in the ATP workflow if the coil height is not adjusted during an ATP workflow. This known defect is alleviated by changing the coil height under the beam properties and then adjusting it back to its original value before optimizing. This defect has been reported to occur less often in the 5.51.11 upgrade, but should it occur, the current solution is still valid. Monaco has four segment generation options for IMRT plans in the ATP adaptation mode. “Original Segments” simply copies the IMRT segment shapes onto the new isocenter location with other no adjustments. “Adapt Segments” adapts to the new beam's eye view using Segment Aperture Morphing (SAM).^8^ “Optimize Weights” adapts the segments using SAM and then reweighs the segments. “Optimize Shapes” expands upon the latter to further allow for adjustments of the segment shapes and weights and the amount of MU per segment. In all cases, dose is recalculated.^9^ In our clinic, it was decided that the “Optimize Shapes” option should be used for all ATP plans. If planning goals cannot be achieved with ATP and deemed clinically unacceptable, then the physician is consulted about following the ATS workflow, which will be addressed in a future report. There were a few high‐ranking failure modes from “Optimize Shapes.” Adapted plans that are calculated on an MR image pulls electron density data from the CT. A high‐ranking failure mode associated with this step is that the electron density could be set incorrectly in the initial reference plan that was done on CT. Another failure mode is that there could be erroneous stray target contours that would deposit dose in unintended areas. Finally, due to a known defect in Monaco at the time of this risk analysis, non‐contiguous structures would cause multi‐leaf collimators (MLCs) to stay open in between detached target volumes in the beam's eye view.

Since there is no recontouring during an ATP workflow, most of these failure modes must be addressed in the initial reference plan check. Electron density (ED) values and the layering of the contours should be reviewed by the planner prior to planning the reference plan and should be second checked by the physicist. The physicist's second check was added to the physicist questionnaire. Similarly, contours should be thoroughly reviewed and cleaned prior to initial planning. This is especially true for volumes that may be non‐contiguous as our clinic does not allow for the treatment of detached targets using the standard ATP workflow. For these cases, “ATS‐lite” is used in which an ATS is performed with all structures excluding the external contour set to propagate rigidly.^10^ On the adapted plan, both planner (dosimetrist or therapist) and physicist should again thoroughly review the isodose lines to check for areas of stray dose or erroneous MLC gaps in the beam's eye view. ED assignments should also be reviewed on the axial, coronal, and sagittal planes. Although there is no option to adjust density assignments in the ATP workflow, the planners should still be trained on the expected electron density range so that they can recognize unusual electron density values in online Monaco. In our clinic, we have noticed that a typical max ED value is 1.8, occurring in the couch model, but this may be higher if the patient has metallic implants. The color of the electron density grid offers a quick review of the assigned densities and makes it easy to catch areas of unexpected high or low densities. Spot checks of the assigned densities is done using the volume cursor.

### 3.5 Plan review and approval

After the plan is nearly finished calculating, a verification scan is taken to double check patient's position for motion during the planning process. The verification scan offers a last opportunity to adjust the plan before treatment in case there was patient motion during the adaptation stage. High‐ranking failure modes associated with the verification scan were forgetting to take the verification scan and not properly evaluating the scan.

The mitigation for forgetting to take a verification scan is already in place with the online checklist and was added to the physicist questionnaire (Figure 1). The online checklist details the most important steps in an ATP workflow and served as the mitigation for multiple other failure modes across the entire ATP workflow.

To address the other failure mode of a poor evaluation of the verification scan, the proposed mitigation is to evaluate the image in a similar manner as the fusion review process. Both planner and physicist should independently inspect the verification scan to ensure that the patient's position has not changed significantly. Like the issue during fusion review, it may be difficult to differentiate between the scans, so both planner and physicist should always double check that the correct image is in view.

### 3.6 Plan checking

After the adaptive plan is approved, the plan information is automatically sent to MOSAIQ for treatment. The high‐ranking failure mode from this step, which scored high for both overall RPN and severity, relates to data transfer issues. If data corruption occurred in the transfer to MOSAIQ, the delivered plan would be incorrect.

To help mitigate this issue, our clinic has implemented an in‐house automated data transfer integrity check script, called Smart Plan Integrity Check (Smart PIC), that compares the data from Monaco with MOSAIQ control point by control point. This script needs to be manually run by the physicist and there is a check box in the ARIA questionnaire to help remind the physicist. The physicist should only allow for beam‐on if Smart PIC passes and investigate failures before proceeding with treatment. Failures may also occur if the wrong Monaco plan is selected for comparison or there is a directory connection issue. If there is a directory connection issue, the plan will not be sent to MOSAIQ, and the machine cannot treat without the plan. In addition to the automated comparison of the plans, an independent MU check is run^11^ on the adapted plan. The independent MU check does a gamma analysis for each field. As an addition safety layer, an additional proposed mitigation is to enhance the secondary MU check script to automatically detect if Smart PIC was run and warn the user running the secondary MU check if Smart PIC has not passed. In cases when the secondary independent check of any field is below passing criteria, a patient‐specific QA (PSQA) measurement is performed using departmental PSQA procedures. Although rare, that QA may be below the passing threshold. If that occurs, the log‐file fluence is analyzed and treatment of the following fraction is paused until additional tests and analysis can be run to discover the problem.

### 3.7 Treatment

During treatment, cine MRI is used to monitor the patient's motion in real time in three orthogonal planes centered on the chosen motion monitoring structure. In the absence of gating, the high‐ranking failure mode from this step is not watching the motion monitor (MM) window and missing clinically significant motion. Since multiple staff could be watching the MM window, there may be a perception that someone else is watching which increases the chance of being distracted. However, it is important that the patient is monitored during beam delivery as large motions could have significant dosimetric consequences.

The mitigation suggested by the risk analysis team is to designate a team member to watch the MM window so that there is clarity in roles regarding patient monitoring. A radiation therapist would be assigned this task before the patient arrives and this person will be explicitly responsible for watching the MM window so that she or he can react should the patient move in an unacceptable manner. This designee should not perform any other duties while the beam is being delivered. In line with this, it was recommended that clearer action levels should be implemented so that a clear procedure exists for how to respond to patient motion rather than relying on subjective criteria for what degree of motion warrants pausing treatment.

### 3.8 Completion plans

**FIGURE 3:**
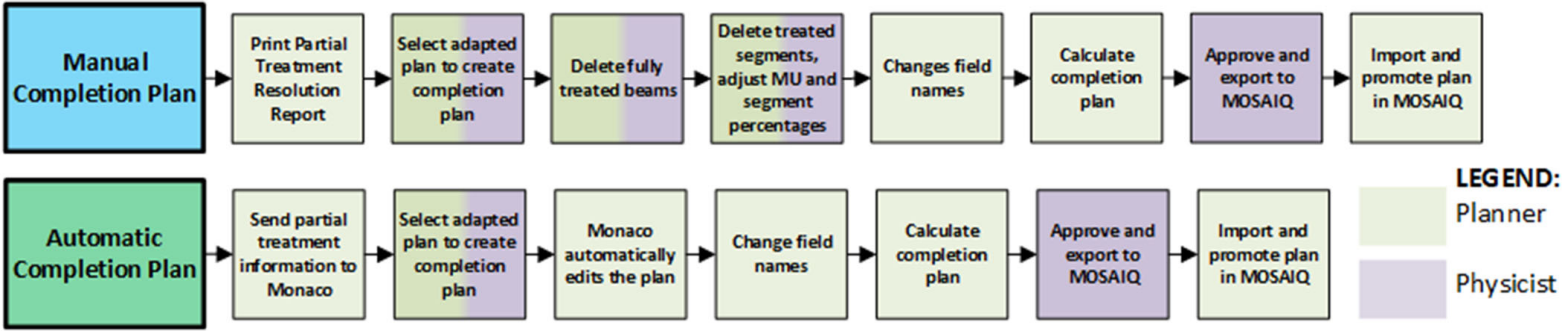
Completion plan process map. The colors of each box indicate the responsible staff member for that step. Multiple colors indicate that multiple staff may be responsible in completing that step.

Although not a part of the standard ATP workflow, completion plan generation is a special case scenario when ATP is employed. In these situations, a partial treatment has occurred, and the remaining dose needs to be delivered later, such that reimaging is necessary. This completion plan is a one fraction copy of the intended treatment plan with only the undelivered segments. Monaco version 5.51.11 and MOSAIQ version 2.83 offers both an automatic and manual generation of a completion plan. Process maps for both completion plan methods are shown in Figure 3.

The automatic completion plan process sends the partially delivered plan to Monaco which is used to help generate the completion plan. In MOSAIQ, there is the option to send the partial treatment information to Monaco and to print a “Partial Treatment Resolution Report” which details the partial delivery and how to edit the plan to keep only the remaining MUs. In Monaco, the user should select the correct adapted plan to create a completion plan. Using the partially delivered plan information, Monaco will automatically delete any fields and segments already delivered. The user then needs to simply update the field names, calculate the dose, approve the plan, and transfer to MOSAIQ.

Many safety features exist already in Monaco and MOSAIQ to help prevent errors that could occur in the completion plan generation process. The “Partial Treatment Resolution Report” states the plan's unique identifier (UID) which the user can use to make sure the correct adapted plan is selected to make the completion plan. In offline Monaco, the user cannot add any beams to completion plans as only the delete option is available, minimizing the chances of making unintended changes. The treatment unit also cannot be changed. The plan can also only be calculated using the specified MUs; the optimization option is not available to the user as reoptimizing would create a different plan that goes to the full dose. Monaco also requires the user to update the field names before calculation or approval could occur. If the user had left the field names the same, the edited fields would override the fields in MOSAIQ for future fractions. This safety feature is new to version 5.51.11, as previous versions did not have this hard stop requirement.

The manual completion plan process has more opportunities for high severity errors. To manually generate a completion plan, the user must delete the fields that have been delivered. For partially delivered fields, the user must update the remaining MU for that field and remove delivered segments. The danger in deleting segment occurs because when a segment is deleted, remaining segments are renumbered. However, the resolution report does not get updated since that is a static document. The user guide's suggested procedure for deleting segments is to delete them in sequential order so that it is easier to keep track of which segments need to be removed.^12^ Incorrect editing of the completion plan is a failure mode that could have severe dosimetric impacts on the treatment. In our clinic, there was a near miss incident where this failure mode occurred. When trying to manually generate a completion plan, segments were deleted from the wrong field. Fortunately, a mitigation for this failure mode is in place within MOSAIQ. When trying to promote the plan to MOSAIQ, there was a plan mismatch error. While there is no built‐in check for the generation of the completion plan, the hard stop from MOSAIQ means that at least the error will not reach the patient.

### 3.9 Evaluation of mitigations

Initial scoring of failure modes did not consider any pre‐existing mitigations or QA processes. After ranking the failure modes and finding mitigations for the top 20%, team members were asked to rescore the high‐ranking failure modes, without referencing previous scores, while considering the mitigations. The idea is to evaluate the effectiveness of the mitigations. The average scores of the high‐ranking failure modes with and without mitigations are displayed in Table 2. The mean scores for each question were compared using a Wilcoxon signed‐rank test to estimate the overall change in each of the categories. Both O and D decreased approximately 19% (*p*‐value < 0.05), while overall RPN decreased approximately 36% (*p*‐value < 0.05). Severity did not see a decrease (*p*‐value 0.76) and the average score stayed the same as the mitigations would not have changed the consequences of the error should it occur.

**TABLE 2:** Initial and rescored O, S, D, and RPN scores for the high‐ranking failure modes

	O	S	D	RPN
Initial scores	1.9	3.0	2.7	16.0
Rescore	1.6	3.0	2.2	10.3

## 4 DISCUSSION

In our clinic, many mitigations for the high‐ranking failure modes are already a part of the daily workflow, even prior to this risk analysis. It is crucial that mitigations do not significantly increase the treatment time, as it increases patient discomfort and the likelihood of motion. We believe that implementing the suggested mitigations should not increase the treatment time significantly. Most of the mitigations occur either automatically, simultaneously with other steps already in the workflow, or should take less than a minute to perform. The largest time addition to the workflow comes only from the independent review of fused images, as we are now doubling the time it takes to review a fusion. We estimate that implementation of the suggested mitigations should take an additional 5 min.

The proposed mitigations for the Unity MRL are similar to those developed for the ViewRay MRL (ViewRay, Cleveland, OH). Prior groups have also utilized checklists to provide guidance throughout the workflow and automated checks for plan consistency and integrity. A secondary monitor unit check and dosimetric measurements of the plan were also performed. Specialized training was also necessary for all professionals treating on the MRL as workflow and the software used likely deviated from those that previously existed in the clinic.^13^, ^14^, ^15^ Klüter et al. also utilized independent review by two parties for critical steps in the workflow, validating the importance of having redundant checks when automated ones are not available. Our mitigation for the Unity MRL employs Klüter et al.’s “four‐eye principle”^13^ in the “call‐out” procedure performed during initial image transfer and the reference plan selection, as well as the independent view of fusion between the daily and reference images and initial and verification images.

Other mitigations suggested for the Unity MRL may be more applicable due to the specific software and settings used by our clinic. During the initial implementation of MOSAIQ, choosing to prevent treatment unless a daily assessment form was filled was a decision made by the group with safety in mind. The assessment in MOSAIQ is customized with questions and prompts that offer the therapists a chance to take a “time‐out” to thoroughly review the paper questionnaire form filled out by the patient. However, filling out the assessment without the patient present ended up being the decision that made the most logistical sense, as the treatment computer is in a separate zone compared to the patient changing room. Similarly, the issue of viewing the wrong MR image when two images are overlaid may not be present in other treatment planning systems, as it is due to the user interface design from Monaco. Our clinic has raised this issue to the Monaco design team and are hopeful for a fix in a future upgrade.

## 5 CONCLUSION

MR‐guided adaptive treatment planning offers patients an exciting prospect for their radiation treatment delivery. Being able to adjust their treatment plan according to day‐to‐day changes in anatomy, which are more easily delineated due to MR imaging, translates to more accurate dose delivery and better sparing of OAR. However, since the adaptive workflow is radically different from a conventional treatment workflow, it is fertile ground for risk analysis which offers the clinic a chance to catch errors before they occur. The adaptive workflow is a very dynamic process, and we always strive to improve patient care quality, treatment time, and patient safety.

## AUTHOR CONTRIBUTIONS

All authors listed for this manuscript actively participated in the risk analysis process for the adapt‐to‐position workflow on the Unity. This included reviewing the process map to ensure no steps were missing and that the level of detail was appropriate, brainstorming failure modes and causes of those failure modes, independently scoring each of the high‐ranking failure modes, and coming up with mitigations for those failure modes.

## CONFLICT OF INTEREST

The authors declare no conflict of interest.

## References

[1] Huq MS , Fraass BA , Dunscombe PB , et al. The report of Task Group 100 of the AAPM: application of risk analysis methods to radiation therapy quality management. Med Phys. 2016;43(7):4209. 10.1118/1.4947547 27370140 PMC4985013

[2] Online repository for TG‐100 resources. https://mpec.aapm.org/repository/home.php

[3] Schuller BW , Burns A , Ceilley EA , et al. Failure mode and effects analysis: a community practice perspective. J Appl Clin Med Phys. 2017;18(6):258‐267. 10.1002/acm2.12190 28944980 PMC5689935

[4] de Fong Los Santos LE , Evans S , Ford EC , et al. Medical Physics Practice Guideline 4.a: development, implementation, use and maintenance of safety checklists. J Appl Clin Med Phys. 2015;16(3):5431. 10.1120/jacmp.v16i3.5431 26103502 PMC5690123

[5] Watson RE Jr , Tesfaldet M , Warren J , Hoff MN . MR imaging safety events: analysis and improvement. Magn Reson Imaging Clin N Am. 2020;28(4):593‐600. 10.1016/j.mric.2020.07.004 33040999

[6] ACR Committee on MR Safety: , Greenberg TD , Hoff MN , et al. ACR guidance document on MR safe practices: updates and critical information 2019. J Magn Reson Imaging. 2020;51(2):331‐338. 10.1002/jmri.26880 31355502

[7] Watson RE Jr , Edmonson HA . MR safety: active implanted electronic devices. Magn Reson Imaging Clin N Am. 2020;28(4):549‐558. 10.1016/j.mric.2020.08.001 33040995

[8] Ahunbay EE , Peng C , Chen GP , et al. An on‐line replanning scheme for interfractional variations. Med Phys. 2008;35(8):3607‐3615. 10.1118/1.2952443 18777921

[9] Winkel D , Bol GH , Kroon PS , et al. Adaptive radiotherapy: the Elekta Unity MR‐linac concept. Clin Transl Radiat Oncol. 2019;18:54‐59. 10.1016/j.ctro.2019.04.001 31341976 PMC6630157

[10] Gupta A , Dunlop A , Mitchell A , et al. Online adaptive radiotherapy for head and neck cancers on the MR linear accelerator: introducing a novel modified adapt‐to‐shape approach. Clin Transl Radiat Oncol. 2021;32:48‐51. 10.1016/j.ctro.2021.11.001 34849412 PMC8608651

[11] Yang J , Zhang P , Tyagi N , et al. Integration of an independent monitor unit check for high‐magnetic‐field MR‐guided radiation therapy system. Front Oncol. 2022;12:747825. 10.3389/fonc.2022.747825 35359395 PMC8963466

[12] Elekta, Ltd . Unity treatment session interruptions. 2021.

[13] Klüter S , Schrenk O , Renkamp CK , et al. A practical implementation of risk management for the clinical introduction of online adaptive magnetic resonance‐guided radiotherapy. Phys Imaging Radiat Oncol. 2021;17:53‐57. 10.1016/j.phro.2020.12.005 33898779 PMC8058032

[14] Cai B , Green OL , Kashani R , Rodriguez VL , Mutic S , Yang D. A practical implementation of physics quality assurance for photon adaptive radiotherapy. Z Med Phys. 2018;28(3):211‐223. 10.1016/j.zemedi.2018.02.002 29550014

[15] Garcia Schüler HI , Pavic M , Mayinger M , et al. Operating procedures, risk management and challenges during implementation of adaptive and non‐adaptive MR‐guided radiotherapy: 1‐year single‐center experience. Radiat Oncol. 2021;16(1):217. 10.1186/s13014-021-01945-9 34775998 PMC8591958

